# SEOM-GEMCAD-TTD clinical guidelines for the management of hepatocarcinoma patients (2023)

**DOI:** 10.1007/s12094-024-03568-4

**Published:** 2024-06-24

**Authors:** Carlos López López, Mariona Calvo, Juan Carlos Cámara, Beatriz García-Paredes, Carlos Gómez-Martin, Ana María López, Roberto Pazo-Cid, Javier Sastre, Ricardo Yaya, Jaime Feliu

**Affiliations:** 1 Medical Oncology Department, H. U. Marqués de Valdecilla, IDIVAL, UNICAN, Santander, Cantabria Spain; 2 Medical Oncology Department, Institut Català d’Oncologia-L’Hospitalet del Llobregat, Barcelona, Spain; 3 Medical Oncology Department, Hospital Universitario Fundación Alcorcón, Madrid, Spain; 4 Medical Oncology Department, Hospital Universitario Clínico San Carlos, Madrid, Spain; 5 Medical Oncology Department, Hospital Universitario 12 de Octubre, Instituto de Investigación Sanitaria Hospital 12 de Octubre (imas12), Madrid, Spain; 6 Medical Oncology Department, Hospital Universitario de Burgos, Burgos, Spain; 7 Medical Oncology Department, Hospital Universitario Miguel Servet, Zaragoza, Spain; 8 Medical Oncology Department, Instituvo Valenciano de Oncología, Valencia, Spain; 9 Medical Oncology Department, Hospital Universitario de La Paz, IdiPAZ, CIBERONC, UAM, Madrid, Spain

**Keywords:** Hepatocellular carcinoma, Guidelines, Diagnostic, Staging, Treatment

## Abstract

Hepatocellular carcinoma (HCC) is the most common primary malignancy in the liver and is the third cause of cancer-related death worldwide. Surveillance with abdominal ultrasound should be offered to individuals at high risk for developing HCC. Accurate diagnosis, staging, and liver function are crucial when determining the optimal therapeutic approach. The BCLC staging system is widely endorsed in Western countries. Managing this pathology requires a multidisciplinary, personalized approach, generally with a multimodal strategy. Surgery remains the only curative option, albeit local and systemic therapy may also increase survival when surgery is not suitable. In advanced disease, systemic treatment should be offered to patients with ECOG/PS 0-1 and Child–Pugh class A.

## Incidence and epidemiology

Hepatocellular carcinoma (HCC) is the sixth most diagnosed cancer and was the third leading cause of cancer mortality worldwide in 2020, with approximately 906,000 new cases and 830,000 deaths. Rates of both incidence and mortality are two to three times higher among men than women in most regions, and liver cancer ranks fifth in terms of global incidence and second in terms of mortality for men [1]. The estimated incidence in Spain for 2023 is some 6695 (2.4%). Given its 4.7/100,000 mortality rate, HCC is the seventh cause of cancer-related death in Spain [2]. Incidence varies depending on geographic location and different specific risk factors. The main risk factors for HCC include chronic hepatitis B virus (HBV) or hepatitis C virus (HCV) infection, heavy alcohol intake, excess body weight, and type 2 diabetes. In most high-risk areas (China, the Republic of Korea, and sub-Saharan Africa), the foremost factors are chronic HBV infection, aflatoxin exposure, or both, whereas in Western Europe, North America, and Japan, HCV infection is he predominant cause [3].

The major risk factors are in flux, considering the declining prevalence of HBV and HCV thanks to vaccine and antiviral treatments [4, 5]. More and more, non-alcoholic fatty liver disease (NAFLD) is found to be increasingly common in people with HCC, especially in developed countries [6] and metabolic syndromes, including diabetes mellitus, hyperlipidemia, and hypertension, all of which often coexist with NAFLD and incur additional risk for HCC development [7].

Cirrhosis of any etiology is the strongest risk factor for HCC (90%). Subjects with cirrhosis resulting from chronic HBV infection have a 100-fold increased risk of developing HCC. The risk of HCC in patients with cirrhosis secondary to HCV is > 2% per year, causing most of the new cases in Europe [3]. A recently published study in individuals with NASDL found that the annual incidence of HCC in NAFLD with cirrhosis is 2.25% [8]. This risk speaks to advocating that all people at high risk for HCC should be entered into a surveillance program.

## Methodology

This guideline is based on a systematic review of relevant published studies and with the consensus of ten treatment expert oncologists from two Spanish digestive cooperative groups—the Grupo Español Multidisciplinar de Cáncer Digestivo (GEMCAD) and Grupo Español de Tratamiento de los Tumores Digestivos (TTD)—the Spanish Society of Medical Oncology (SEOM), and an external review panel comprised of two experts designated by the SEOM. The Infectious Diseases Society of America–US Public Health Service Grading System for Ranking Recommendations in Clinical Guidelines has been used to assign levels of evidence and grades of recommendation [9] (Table 1).

**Table 1:** Levels of evidence and grades of recommendation

Levels of evidence
I. Evidence from at least one large randomized, controlled trial of sound methodological quality (low potential for bias) or meta-analyses of well-conducted randomized trials without heterogeneity
II. Small randomized trials or large randomized trials with a suspicion of bias (lower methodological quality) or meta-analyses of such trials or of trials with proven heterogeneity
III. Prospective cohort studies
IV. Retrospective cohort studies or case–control studies
V. Studies without control group, case reports, expert opinions

Grades of recommendation
A. Strong evidence for efficacy with a substantial clinical benefit; strongly recommended
B. Strong or moderate evidence for efficacy, but with limited clinical benefit; generally recommended
C. Insufficient evidence of efficacy or benefit does not outweigh the risk or the disadvantages (adverse events, costs,); optional
D. Moderate evidence against efficacy or for adverse outcome; generally, not recommended
E. Strong evidence against efficacy or for adverse outcome; never recommended

## Diagnosis, pathology, and molecular biology

### Surveillance

Individuals at high risk for HCC should be monitored: chronic HBV infection with high-risk features (high viral load, Asian or African ancestry, family history, etc.) and those with cirrhosis of any etiology in Child–Pugh stage A or B. Only patients with Child–Pugh stage C awaiting liver transplantation benefit from surveillance. There is no consensus regarding the value of surveillance in patients with post-sustained virologic response (post-SVR) HCV infection without cirrhosis or in those with NAFLD without cirrhosis [10,11].

Abdominal ultrasound (US) every 6 months remains the reference test for surveillance [12]. Alpha-fetoprotein (AFP) detection is slightly more sensitive and is cost-effective [13, 14], although there is no consensus concerning its use as a screening method [15, 16].

### Diagnosis

Lesions ≥ 1 cm in size and specific imaging criteria (arterial phase hyperenhancement and washout on portal venous or delayed phases of contrast-enhanced multiphase CT or MRI) can be regarded as HCC without histologic confirmation in patients with cirrhosis of the liver. MRI has demonstrated somewhat greater sensitivity than and similar specificity as CT [17]. The Liver Imaging Reporting and Data System (LI-RADS) is the most widely accepted system to standardize and increase HCC diagnostic accuracy [18]. Serum biomarkers alone, including AFP, do not have sufficient accuracy to establish the diagnosis.

Most lesions < 1 cm are not HCC. Closer follow-up with US every three months for 24 months is recommended. If the lesion remains stable, it is safe to return to semi-annual surveillance. If the lesion grows, the aforementioned diagnostic algorithm should be applied.

In the absence of cirrhosis and in those lesions classified as “probably malignant” but lacking specific imaging criteria for HCC, histological confirmation by biopsy is strongly recommended.

### Pathology and molecular biology

The histological classification and diagnostic criteria for HCC have been defined by WHO and the International Consensus Group for Hepatocellular Neoplasia [19, 20]. Immunohistochemistry biomarkers such as glypican 3, heat shock protein 70, and glutamine synthetase are used to enhance diagnostic specificity to up to 100% with the presence of two or more of these markers [21, 22]. Approximately 80% of HCC cases arise in cirrhotic livers and different subtypes have been associated with specific molecular and cytogenetic abnormalities: the scirrhous subtype with TSC1/TSC2 mutations, the steatohepatitic subtype with frequent IL6/JAK/STAT activation, the macrotrabecular massive subtype possessed TP53 mutation and FGF19 amplification, and the DNAJB1-PRKACA fusion gene is pathognomonic for the fibrolamellar subtype [23].

The molecular landscape of HCC comprises a convergence of genetic, epigenetic, and signaling dysregulations. Several cancer driver genes with oncogenic or tumor suppressive functions recurrently altered in HCC have been identified. Telomerase activation is the most prevalent one by means of TERT promoter mutations, viral insertions, chromosome translocation, or gene amplification that are observed in up to an 80% of HCC [24, 25]. Activation of the Wnt/β-catenin signaling pathway caused by mutations in CTNNB1, AXIN1, or APC inactivation is found in 30–50% of cases. Usual mutations or genetic alterations related with cell cycle control are detected in TP53, RB1, CCNA2, CCNE1, PTEN, ARID1A, ARID2, RPS6KA3, or NFE2L2 genes. Furthermore, recurrent focal chromosome amplifications in CCND1, FGF19, VEGFA, MYC, or MET genes, as well as changes in epigenetic regulation, oxidative stress, and the AKT–mTOR and MAPK pathways have been reported in HCC [26, 27].

Multiomic analyses have classified HCC based on genetic, metabolic, immune, and chromosomal profiles. Many groups have reported molecular classification of HCC based on genomic profiles [28–31] that can ultimately be grouped into two major molecular types [32]. One (proliferation class) is broadly characterized by signal enhancement related to cell proliferation and cell cycle progression and is generally associated with a more aggressive phenotype. The second class (non-proliferation class) accounts for 50% of HCC and is similarly subdivided into two groups: one characterized by activation of the WNT/β-catenin signaling pathway because of frequent CTNNB1 mutations and the other (interferon subclass) with an activated IL6–JAK–STAT signaling pathway and a more inflamed tumor microenvironment with frequent TERT promoter mutations.

### Recommendations

- Surveillance should be offered to high-risk patients if liver function and comorbidities allow for active treatment (I, A).
- Abdominal US every 6 months is the most appropriate screening technique (II, A).
- Multiphasic CT or dynamic contrast-enhanced MRI enables a non-invasive radiological diagnosis to be established in cirrhotic patients with specific imaging criteria (II, A).
- In the absence of cirrhosis or at-risk chronic HBV infection, HCC diagnosis should be confirmed by biopsy (I, A).
- Pathological diagnosis of HCC should be based on the International Consensus recommendations using the required histological and IHC analyses (V, A).
- Histological analysis enables molecular characterization and identification of potential therapeutic targets to be made (IV, B).

## Staging and risk assessment

Recent progress in the field of HCC research have led to staging systems and molecular classifications being developed, thereby providing valuable tools by which to predict prognosis and inform treatment decisions.

**Fig. 1:**
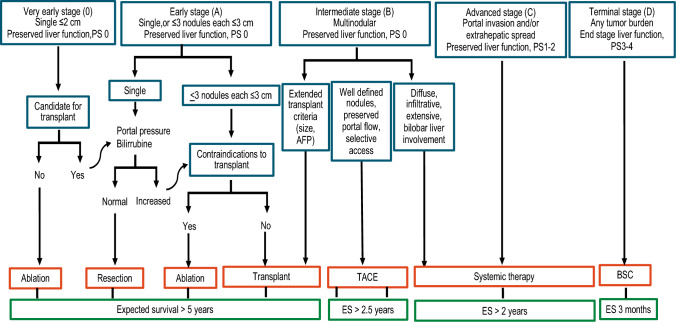
Modified from Reig M. et al. J Hepatol. 2022 Mar;76(3):681–693. BCLC staging and treatment strategy for HCC in 2022. The BCLC system establishes prognosis according to the five stages linked to first-line treatment recommendations. The expected outcome is expressed as median survival of each HCC stage based on the available scientific evidence. Liver function should be evaluated beyond the conventional Child–Pugh staging. *AFP* alpha-fetoprotein, *ALBI* albumin-bilirubin, *BCLC* Barcelona Clinic Liver Cancer, *BSC* best supportive care, *ECOG-PS* Eastern Cooperative Oncology Group-performance status, *LT* liver transplantation, *MELD* model of end-stage liver disease, *TACE* transarterial chemoembolization

Staging systems, such as the widely endorsed and recently updated Barcelona Clinic Liver Cancer Staging System (BCLC) [33], take the tumor burden and underlying hepatic injury into account to predict prognosis in HCC (Fig. 1). Factors such as tumor characteristics, liver function status, and the patient's overall medical condition are likewise factored in. However, despite the availability of several staging systems [34], no one is universally accepted, because of variations in population cohorts and the diverse etiology of HCC across different geographic regions [35].

**Fig. 2:**
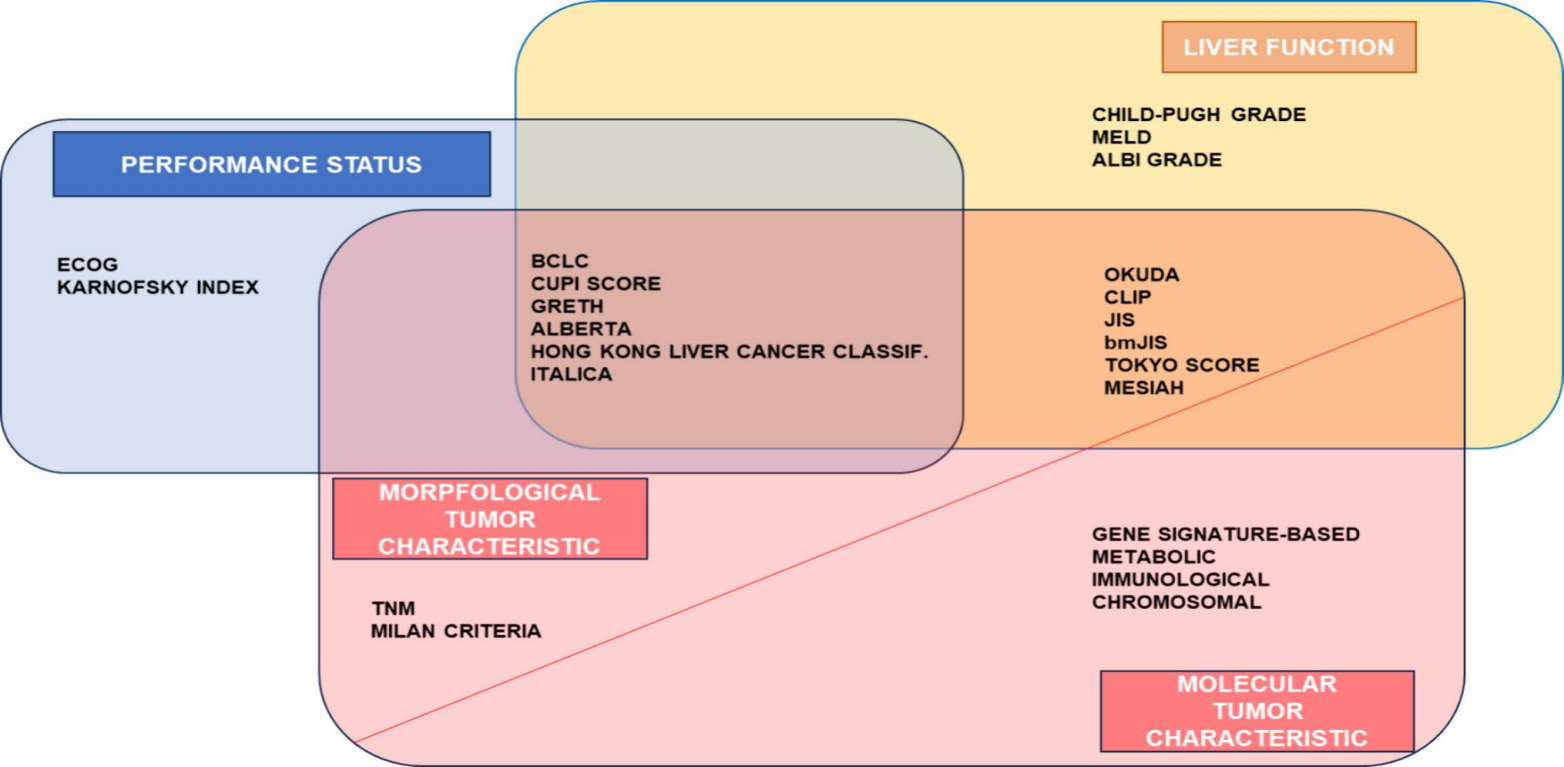
Modified from Addissie et al. (Clin Liver Dis. 2015; 19: 277–294), Sastre et al. Clin Transl Oncol. (2015; 17:988–995) and Chidambaranathan-Reghupaty (Adv Cancer Res. 2021; 149: 1–61). *ECOG* Eastern Cooperative Oncology Group, *BCLC* Barcelona Clinic Liver Cancer, *CUPI SCORE* Chinese University Prognostic Index, *GRETCH* Groupe d’Etude et de Traitement du Carcinome Hepatocellulaire, *MELD* model for end-stage liver disease, *ALBI*, albumin-bilirubin, *OKUDA* OKUDA staging system, *CLIP* Cancer of the Liver Italian Program, *JIS* Japanese integrated staging, *bm-JIS* biomarker combined JIS, *TNM* tumor-node-metastasis staging, *MESIAH* Model to Estimate Survival In Ambulatory HCC patients score, *ITA.LI.CA* Italian Liver Cancer score

On the other hand, molecular classifications of HCC [36] have been proposed that provide a deeper understanding of tumor biology and mechanisms of carcinogenesis. Furthermore, immune profile-based classifications have associated certain subgroups with specific molecular profiles (Fig. 2).

Validating these gene-based classification systems in metabolic and immune-based contexts show promise, particularly with respect to their prognostic value. That being said, further validation is needed and efforts should be made to include larger, independent cohorts to ensure their generalizability and clinical utility.

Despite the inroads made, updates and refinements of both staging and molecular classification systems are still needed. Recent data on therapies, transplantation criteria, and viral hepatitis treatment must be incorporated into such revisions. Ongoing developments in HCC research and treatment should continue to drive the constant updating of these systems.

### Recommendations

- Staging systems play a crucial role in predicting the prognosis of HCC. The BCLC system is recommended for this purpose (I, A).
- Molecular classifications can provide prognostic and therapeutic information, although they must be refined and validated (V, C).

## Management of local and locoregional disease

The management of local/locoregional disease demands a multidisciplinary approach, usually with a multimodal treatment strategy based on individual patient characteristics, liver function, and tumor stage.

### Local disease (early-stage HCC, BCLC-0, and BCLC-A)

- Anatomical surgical resection (SR) is the gold standard for early-stage HCC, especially in subjects with preserved liver function (Child–Pugh class A), no major vascular invasion, no portal hypertension (hepatic venous pressure gradient < 11 mmHg, platelet count > 100,000), and a solitary tumor (BCLC stage 0 or A and selected BCLC stage B). [37] Expected SR perioperative mortality is in the range of 2–3%. An anticipated liver remnant of at least 20% in non-cirrhotic patients and at least 30–40% in individuals with cirrhosis 30–40% is recommended. Preoperative portal vein embolization (PVE) can result in 8–27% increased future liver remnant volume with a morbidity rate of 2.2% and 0% mortality. The associating liver partition with portal vein ligation for staged hepatectomy (ALPPS) approach is not recommended (68% morbidity rate, 12% mortality rate). Laparoscopic SR has demonstrated superiority over open SR as it is significantly correlated with fewer postoperative complications with no differences in tumor recurrence and overall survival (OS) [38].
- Ablative therapies (AT) are minimally invasive techniques that seek to destroy the tumor by applying heat (radiofrequency ablation—RFA—and microwave ablation—MWA) or injecting ethanol (PEI) directly into the tumor. These treatments are typically reserved for individuals with small tumors (< 3 cm) and limited liver function. A meta-analysis revealed that RFA has the maximum benefit in terms of OS and recurrence-free survival (RFS) compared to resection in Child–Pugh A class, single-nodule tumors < 2 cm, and AFP < 20 ng/mL [39]. RFA has certain limitations in cases in which tumors are located close to other organs or large vessels. In these situations (10–15%), PEI is recommended. The post-AT recurrence rates are similar to post-SR and may be as high as 80% at 5 years.
- Following SR and AT, early HCC has a high recurrence rate (50% within 2 years, 70–80% within 5 years). Adjuvant treatments (i.e., sorafenib) have failed to improve outcomes and observation is the standard of care [40] .An interim analysis of the phase III IMbrave-050 trial met its primary endpoint; i.e., the combination of atezolizumab and bevacizumab significantly increased RFS compared to active surveillance when used as adjuvant therapy following SR or AT [41]. A longer follow-up is required to evaluate OS. Four other ongoing, phase III clinical trials (CheckMate-9DX, KEYNOTE-937, EMERALD-2, and JUPITER-04) will shed light on the role of adjuvant ICI in HCC.
- Liver transplantation (LT) offers a curative option for patients with HCC and underlying liver cirrhosis. LT is limited by the scarcity of donor organs and strict selection criteria. Patients within Milan criteria (MC) (single HCC nodule < 5 cm or up to 3 nodules < 3 cm each, with no macrovascular involvement, and no extrahepatic disease) could be considered for LT, achieving a 5-year overall survival of more than 70% and 5-year recurrence rate of < 10%. Perioperative mortality and 1-year mortality are expected to be approximately 3% and < 10%, respectively. Bridge or downstaging strategies could be contemplated in selected cases, if waiting list for LT exceeds six months.

Individuals meeting the University of California San Francisco (UCSF) extended criteria for LT (a single nodule up to 6.5 cm or up to three lesions, the largest of which is ≥4.5 cm, with the sum of the diameters ≥8 cm) should receive neoadjuvant treatments or ‘“bridging therapies” to downstaging tumors to MC for LT. Retrospective multi-center data showed that LT was performed after successful downstaging in 58% of the cases, yielding a 5-year post-transplant survival rate of 80%. This indication requires prospective validation. [42]

### Locoregional disease (intermediate stage HCC, BCLC-B)

**Fig. 3:**
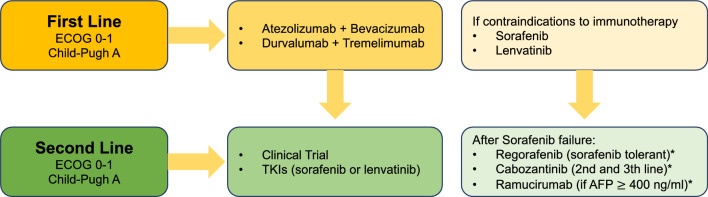
Systemic therapy algorithm for patients with advanced HCC. *Not funded by the Spanish National Health System

The tumor burden in intermediate stage HCC may vary considerably; the disease may be confined to one or two liver segments or be multi-lobar and widespread, and outcomes are highly heterogeneous (OS = 5–25 months). [43, 44] These patients have disease beyond resectability or Milan LT criteria, but have no macrovascular invasion, metastasis, or impaired liver function (Fig. 3).

According to APPLE Consensus Statements, tumors < 7 cm and < 7 lesions are classified into the good response to TACE subgroup (*‘up to seven’* rule), tumors > 6 cm and ≤ 6 lesions or patients ≥ 7 lesions are classified into the poor response to TACE subgroup and could be candidates to DEB-TACE/ systemic treatment. [45]

- Transarterial chemoembolization (TACE) involves the selective administration of chemotherapy agents (doxorubicin or cisplatin) directly into the hepatic artery that supplies the tumor, mixed with embolic agents (i.e., lipiodol), which occlude the tumor’s blood vessels, and with or without a procoagulant material, leading to tumor necrosis. Non-absorbable embolic microspheres charged with cytotoxic agents (DEB-TACE) have been developed. [46] TACE has been shown to improve survival and is recommended as first-line treatment for patients with asymptomatic (ECOG PS 0), large or multifocal, intermediate-stage HCC (BCLC-B) with normal hepatic function (Child–Pugh < 8), and without vascular invasion or extrahepatic spread. Repeating TACE has been acknowledged to prolong OS, but switching to other therapeutic options in the absence of response after two sessions is recommended. In this sense, repeated treatments with RFA, hepatectomy, and TACE can compromise liver function in many patients, ultimately resulting in tumors that are not amenable to treatment with systemic therapy.
- Radioembolization, also known as selective internal radiation therapy (SIRT) and transarterial radioembolization (TARE), involves the intra-arterial injection of radioactive microspheres loaded with β-emitting yttrium-90 isotope into the hepatic artery. These microspheres deliver a high dose of radiation to the tumor, while sparing the surrounding healthy liver tissue, potentially achieving radiation doses that are higher than those achievable with external beam radiation (EBRT). In contrast to TACE, TARE has minimal embolic effects in the hepatic artery distribution and can, therefore, be used in patients with portal vein thrombosis or tumor invasion. TARE is primarily used for subjects with unresectable HCC who have microvascular invasion, excellent liver function, and no extrahepatic disease. A meta-analysis demonstrated no statistically significant difference in survival between TACE and TARE. [47]
- External beam radiation (EBRT) uses intensity-modulated RT (IMRT) or image-guided stereotactic body RT (SBRT). It is typically reserved for individuals with unresectable HCC who have failed or are unsuitable for SR and AT and have no extrahepatic disease, limited tumor burden, and relatively well-preserved liver function.
- Multimodal approaches: a combination of local and locoregional therapies may be used to achieve optimal tumor control. TACE can be combined with RFA or MWA. These strategies can be used in patients with early-stage HCC and contraindications for radical therapies, and prior to liver transplants in patients who are estimated to have a long waiting time. Recently, preliminary favorable data have also been reported pointing toward significant progression-free survival (PFS) benefit by adding systemic therapy to TACE. In fact, clinical trials are currently ongoing attempting to confirm these results.

### Recommendations

- Managing these patients demands a multidisciplinary, personalized approach, with a multimodal strategy (III, A).
- Anatomical surgical resection (SR) is the gold standard for early-stage HCC (BCLC-0 and BCLC-A) (I, A). A liver remnant of at least 20% in non-cirrhotic patients and 30–40% in patients with cirrhosis is recommended (II, B).
- Ablative therapies (RFA or MWA) are reserved for subjects with small tumors (< 3 cm) and limited liver function (II, A). RFA has some limitations in certain cases. In these situations, PEI is recommended (I, A).
- Patients meeting Milan criteria (MC) could be considered for LT (II, A). Bridge or downstaging strategies might be considered if the waiting list for LT exceeds 6 months (II, B).
- TACE is recommended as a first-line treatment for individuals with ECOG 0, intermediate-stage HCC (BCLC-B), and normal hepatic function (II, A). Repeating TACE prolongs OS, but switching to other options in the absence of response after two sessions is recommended (II, B).

## Management of advanced and metastatic disease

Systemic treatment should be offered to patients with diagnosis of stage BCLC-C HCC and those initially BCLC-A or B not candidates or after failure to surgery and/ or loco-regional therapies (advanced HCC).

Until de beginning of the twenty-first century, limited medical management was available for these cases, due to patients’ poor tolerance and the resistance of the disease to chemotherapy. Antiangiogenic therapy and, more recently, immunotherapy have changed the natural course of the disease in these subjects, and response and survival rates have improved dramatically.

It must be remembered that all phase III clinical trials conducted with currently approved drugs and combinations, have only included participants with good liver function (Child–Pugh A). All data from patients treated with liver function CP-B come from real-world data or single/cooperative group series. Patients with ECOG 3 or higher are classified as BCLC-D and have been systematically excluded from clinical trials. There is no scientific support/evidence that endorses treatment for these patients and only palliative care is recommended. [33]

Patient characteristics and comorbidities should be carefully documented prior to any treatment decision, paying special attention to certain medical conditions, such as hypertension, esophageal varicose veins, any recent bleeding, active or prior autoimmune disease or immunodeficiency, previous organ transplant, immunosuppressant medication, HIV infection, active HBV or HCV infection. Likewise, absolute and relative contraindications to immune checkpoint inhibitors (ICIs), antiangiogenics, and multi-TKIs should be considered.

### First Line (Table 2)

**Table 2:** Summary of pivotal trials of HCC systemic therapies (approved by FDA/ EMA)

Study	N	Study arms	OS (months)	PFS/TTP (months)	ORR (%)
First line					
Immunotherapy combinations					
IMbrave 150 [48]	501	Atezolizumab + Bevacizumab	19.2	6.9 (PFS)	27.3
		Sorafenib	13.4	4.3	11.9
HIMALAYA [51]	1171	Durvalumab + Tremelimumab	16.43	5.4 (TTP)	20.1
		Durvalumab	16.56	3.8	17
		Sorafenib	13.77	5.6	5.1
Tyrosine kinase inhibitors (TKIs)					
SHARP [53]	602	Sorafenib	10.7	5.5 (TTP)	2
		Placebo	7.9	2.8	1
Asia-Pacific [54]	226	Sorafenib	6.5	2.8 (TTP)	3.3
		Placebo	4.2	1.4	1.1
REFLECT [55]	954	Lenvatinib	13.6	7.3 (PFS)	18.8
		Sorafenib	12.3	3.6	6.5
Second and subsequent lines (all after sorafenib)					
Tyrosine kinase inhibitors (TKIs)					
RESORCE [56]	573	Regorafenib*	10.6	3.1 (PFS)	11
		Placebo	7.8	1.5	4
CELESTIAL [57]	707	Cabozantinib*	10.2	5.2 (PFS)	4
		Placebo	8.0	1.9	< 1%
Antiangiogenic antibodies					
REACH-2 [58]	292	Ramucirumab*	8.5	2.8 (PFS)	4.6
		Placebo	7.3	1.6	1.1

*Not funded by the Spanish National Healthcare System

#### Immunotherapy plus antiangiogenic combination

Antiangiogenic inhibition is crucial in HCC tumor control and several single-agent phase III trials have demonstrated enhanced overall survival. Recently, a synergistic effect between VEGF inhibition and PD-1/ PDL-1 inhibitors has been proven in several clinical trials.

The combination of intravenous bevacizumab (15 mg/Kg) plus atezolizumab (1200 mg) every three weeks is currently approved advanced HCC. The IMbrave-150 phase III trial comparing this combination against sorafenib revealed significantly greater overall survival (OS) (HR 0.58, *p* < 0.001), longer PFS (HR 0.59, *p* < 0.001), and better response rates (27.3% vs 11.9%, *p* < 0.001) [48]. Combination therapy also prolonged time to deterioration as per patient-reported quality of life and functioning than sorafenib; furthermore, the treatment was well tolerated and hypertension was the most common grade 3 or 4 treatment-related adverse event.

The Asian ORIENT-32 phase II–III study comparing the PD-1 inhibitor sintilimab (200 mg) plus bevacizumab biosimilar (15 mg/Kg) every 3 weeks *versus* sorafenib confirmed the advantage of these types of combinations (HR 0.57, *p* < 0.0001) for OS, as well as for PFS (HR 0.56, *p* < 0.001) and time to deterioration in quality of life [49]. The PD-1 inhibitor camrelizumab has been associated with the VEGFR inhibitor rivoceranib and this combination has evinced better PFS (HR 0.52, *p* < 0.0001) and OS (HR 0.62, *p* < 0.0001) compared to sorafenib. [50] Neither sintilimab or camrelizumab have been approved by the Spanish healthcare authorities.

#### Check-point inhibitor combinations

Programmed cell death receptor-1 and ligand-1 (PD-1/ PD-L1) and cytotoxic T-lymphocyte-associated antigen-4 (CTLA-4) operate via complementary immunosuppressive signaling pathways, and a combined regimen to inhibit both pathways may improve outcomes in cases of advanced HCC. A recent phase III trial combining a single high-dose of tremelimumab (an anti-CTLA-4, 300 mg) plus durvalumab (anti-PD-L1, 1500 mg every 4 weeks) (STRIDE regimen) resulted in significantly prolonged mOS relative to sorafenib (HR 0.78, *p* = 0.0035). Median PFS was not significantly different. In this trial, durvalumab monotherapy was not inferior to sorafenib [51]. This combination has already been approved by the European Medicines Agency (EMA) and also recently by the Spanish healthcare authorities.

The phase I/II trial has yielded promising outcomes with the combination of ipilimumab (CTLA-4 inhibitor) plus nivolumab (anti-PD-1) in different doses and schedules [52]. Final results of the Checkmate 9DW phase III trial comparing the above-named agents to sorafenib or lenvatinib have yet to be published.

#### Tyrosine kinase inhibitors (TKIs)

Some patients with advanced HCC may not be suitable candidates for first-line immunotherapy. In these cases, sorafenib or lenvatinib (oral multikinase inhibitors approved for first-line treatment) could still be considered.

Sorafenib (400 mg bid) was evaluated in two randomized, placebo-controlled, phase III trials (SHARP and Asia–Pacific trials) [53, 54] for the treatment of advanced HCC. Median OS was significantly better in the sorafenib arm in both trials: 10.7 versus 7.9; HR, 0.69; 95% CI, 0.55–0.87; *p* < 0.001 in the SHARP trial. The magnitude of benefit was similar in the Asian study (HR, 0.68; CI, 0.50–0.93; *p* = 0.014), although the mOS was strikingly lower in both arms (6.5 vs. 4.2 months).

In the phase III randomized REFLECT trial [55], lenvatinib (12 mg/day if $\ge$ 60 kg or 8 mg/day if < 60 kg) proved to be non-inferior to sorafenib in first-line treatment, with mOS of 13.6 versus 12.3 months, respectively (HR, 0.92; 95% CI, 0.79–1.06). Moreover, the objective response rate (ORR) by RECIST 1.1 (18.8% vs. 6.5%), time to progression (TTP) (7.4 vs. 3.7 months), and PFS (7.4 vs. 3.7 months) were better with lenvatinib. The safety profile of both drugs differs slightly: weight loss, vomiting, proteinuria, and hypertension are more common with lenvatinib *versus* hand–foot skin reaction, rash, diarrhea, and alopecia in the case of sorafenib. The REFLECT protocol did not include patients with main portal vein invasion or extensive ($\ge$ 50%) hepatic tumor involvement.

### Second and subsequent lines (Table 2)

Approximately one-third of individuals with advanced HCC are eligible for second-line therapy. The decision to switch from first- to second-line treatment should take progression at imaging, pattern of progression, general conditions, and liver function into account.

For subjects who progress on sorafenib, regorafenib 160 mg daily for 3 weeks of each 4-week cycle (only for sorafenib-tolerant patients), cabozantinib 60 mg daily (irrespective of tolerance to sorafenib), and ramucirumab 8 mg/ kg every 2 weeks (if AFP level is > 400 ng/dl, also irrespective of tolerance to sorafenib), have been shown to prolong survival compared to placebo in three randomized, double-blind, placebo-controlled, international phase III trials (RESORCE [56], CELESTIAL [57], and REACH-2 [58], respectively). Unfortunately, none of these drugs are currently funded by the Spanish Public Healthcare System. In addition, there are not head-to-head comparative data between them and they have not been tested after immunotherapy combinations, which are currently a standard of care as first-line treatment for some patients, so evidence-based recommendations cannot be made. Moreover, sorafenib and lenvatinib should be evaluated for consideration as possible effective second-line options in this setting. There is a rationale for offering a multikinase inhibitor after first-line immunotherapy combinations, given the evidence available concerning these drugs in first- and in second-line treatment. Thus, although based on retrospective series and real-world data, some clinical guidelines suggest these drugs as valid alternatives in this clinical context [59, 60]. Hopefully, some of the ongoing studies may shed light on the best way to sequence currently available drugs.

### Recommendations

- Systemic treatment should be offered to patients with stage BCLC-C HCC and those initially BCLC-A or B after failure to locoregional therapies or contraindications for those treatments (I, A).
- Atezolizumab/bevacizumab or durvalumab/tremelimumab are the preferred options for naive patients with ECOG PS 0–1 and Child–Pugh class A (I, A).
- Sorafenib or lenvatinib are first-line alternatives when immunotherapy is contraindicated (I, A).
- Despite the scientific evidence available (I, A) demonstrating an overall survival benefit after first-line sorafenib, neither regorafenib, cabozantinib, nor ramucirumab are funded by the Spanish National Healthcare System.
- Due to the lack of scientific evidence after atezolizumab/bevacizumab, durvalumab/tremelimumab, or lenvatinib, the choice of a second-line agent in this context should be based on each patient’s clinical characteristics, as well as the drug’s toxicity profile and availability (V, C), offering the patient the opportunity to participate in a clinical trial whenever available.
- The use of a multikinase inhibitor (sorafenib or lenvatinib) can be justified after first-line immunotherapy combinations (V, C).

## Follow-up, long-term implications and survivorship

Post-treatment follow-up plays an essential role in cancer care to assess therapeutic efficacy, detect recurrences early, and identify long-term treatment-related complications.

In contrast to the robust evidence supporting primary HCC screening in patients with chronic liver disease, there is insufficient evidence to inform the optimal frequency and modality of HCC surveillance after curative treatment. Therefore, recommendations are based on the general opinion that early identification of disease recurrence may facilitate more therapeutic options and prolong survival.

HCC recurrence after resection has been classified as early recurrence when it appears within 2 years (thought to be due to micro-metastases) and late recurrence if it occurs after 2 years (considered to be de novo lesions) [61]. Furthermore, the risk of HCC recurrence varies depending on the stage, underlying risk factors, and the person’s remnant liver function; it is, therefore, difficult to put forth a uniform recommendation.

Following curative treatment, multiphasic contrast-enhanced CT or MRI should be performed every 3–6 months for 2 years, and then every 6 months [62]. In addition, if initially elevated, AFP should be determined every 3–6 months for the first 2 years and every 6 months thereafter.

Individuals with more advanced stages of disease who are treated with TACE or systemic agents, can be evaluated by dynamic CT or MRI every 2–3 months to guide therapy decisions. AFP may be useful in assessing response to treatment when the level is high at diagnosis. Nevertheless, tumor markers cannot replace imaging studies [63].

Follow-up should also include regular physical examination and liver function evaluation in all patients.

As the number of HCC survivors is increasing thanks to better early detection and more effective therapies, a considerable number of them may experience late and/or long-term physical and psychosocial effects, which will impact their quality of life. During follow-up, these problems must be identified and treated, in addition to recommending a healthy lifestyle.

### Recommendations

- After curative treatment, dynamic CT or MRI may be recommended every 3–6 months for 2 years, then every 6 months. (Level of evidence III. Grade of recommendation A).
- Advanced stage patients can be evaluated by dynamic CT or MRI every 2–3 months (Level of evidence III. Grade of recommendation A).

## Data Availability

Not Applicable.
